# Cognitive dysfunction 1 year after COVID‐19: evidence from eye tracking

**DOI:** 10.1002/acn3.51675

**Published:** 2022-10-20

**Authors:** Federico Carbone, Laura Zamarian, Verena Rass, Stefanie Bair, Marcel Ritter, Ronny Beer, Philipp Mahlknecht, Beatrice Heim, Victoria Limmert, Marina Peball, Philipp Ellmerer, Alois Josef Schiefecker, Mario Kofler, Anna Lindner, Bettina Pfausler, Lauma Putnina, Philipp Kindl, Judith Löffler‐Ragg, Stefan Kiechl, Klaus Seppi, Atbin Djamshidian, Raimund Helbok

**Affiliations:** ^1^ Department of Neurology Medical University of Innsbruck 6020 Innsbruck Austria; ^2^ Interactive Graphics and Simulation Group University of Innsbruck 6020 Innsbruck Austria; ^3^ Department of Internal Medicine II Medical University of Innsbruck 6020 Innsbruck Austria

## Abstract

Increasing evidence suggests persistent cognitive dysfunction after COVID‐19. In this cross‐sectional study, frontal lobe function was assessed 12 months after the acute phase of the disease, using tailored eye tracking assessments. Individuals who recovered from COVID‐19 made significantly more errors in all eye tracking tasks compared to age/sex‐matched healthy controls. Furthermore, patients who were treated as inpatients performed worse compared to outpatients and controls. Our results show impaired inhibitory cortical control in individuals who recovered from COVID‐19. The association between disease severity and its sequelae may contribute to a better understanding of post‐COVID‐19 cognitive function.

## Introduction

COVID‐19 is caused by the severe acute respiratory syndrome coronavirus 2 which, similarly to other known coronaviruses, leads primarily to respiratory symptoms. However, a considerable number of patients with COVID‐19 also develop neurological, neuropsychiatric, and neuropsychological symptoms during the acute phase, lasting even months after recovery.^1^ Particularly, cognitive deficits such as impairment of attention, memory and executive function have been described.^2^, ^3^, ^4^ A recent multicentre study showed persistent cognitive impairment in about 17% of COVID‐19 patients 6 months after hospitalization.^5^ Other frequently reported symptoms include fatigue, headache, olfactory impairment, depression, and anxiety.^6^ So far, the literature is scarce on long‐term neuropsychological complications of COVID‐19 patients.^7^

One important phenotype reported in literature refers to neurocognitive deficits of frontal cortical function.^8^ We aimed to assess mental flexibility and inhibitory control using tailored eye‐tracking tasks in COVID‐19 patients 12 months after disease onset. We hypothesized that individuals who had COVID‐19 would perform significantly worse than healthy controls (HC) on all saccadic paradigms.

## Methods

### Participants

This was a cross‐sectional controlled study. Between April and October 2021, we recruited 55 patients 1 year after a PCR confirmed diagnosis of COVID‐19, who were part of a prospective observational study.^9^ Saccadic performance was compared to 23 non‐depressed, age‐matched HC without a history of COVID‐19 infection. Participants provided written informed consent according to the declaration of Helsinki. The study was approved by the local ethic committee (Medical University of Innsbruck, EK Nr. 1103/2020) and registered on ClinicalTrials.gov (NCT05025839).

### Experimental protocol

Patients were assessed for mental and physical fatigue, as well as for symptoms of depression and anxiety using dedicated questionnaires. Cognition was also tested (Table 1). We assessed the presence of persistent symptoms 1 year after COVID‐19 including fatigue, sleep disturbances, impairment in concentration, headache, and forgetfulness.

**Table 1:** Demographic data of study population.

	Demographic data of study population		*p* value
	Healthy controls (*n* = 23)	Covid‐19 Patients (*n* = 55)	
Sex M (%)	43.5	56.3	0.305
Age (years)	57.1 ± 8.6	54.4 ± 13.3	0.387
MoCA	28.0 ± 1.3	27.1 ± 2.5	0.205
Education (years)	14.2 ± 2.2	13.3 ± 2.5	0.145
HADS‐A		5.3 ± 3.8	
HADS‐D		3.4 ± 3.7	
FAS		21.9 ± 6.6	

	Sub‐analysis of COVID‐19 patients		*p* value
	Outpatients (*n* = 17)	Inpatients (*n* = 38)	
Sex M (%)	23.6	71.0	**0.001**
Age (years)	46.3 ± 12.9	58.1 ± 11.8	**0.003**
MoCA	28.43 ± 1.31	26.5 ± 2.7	**0.013**
Education (years)	14.4 ± 2.5	12.7 ± 2.4	0.104
Days since diagnosis	419.9 ± 28.8	418.8 ± 24.6	0.886
FAS	18.5 ± 6.9	22.1 ± 5.7	0.100
HADS‐A	4.8 ± 4.5	5.4 ± 3.4	0.613
HADS‐D	2.6 ± 3.8	3.8 ± 3.6	0.304
Persistent fatigue^1^ (%)	41.2	36.8	0.805
Persistent concentration impairment^1^ (%)	35.3	23.7	0.402
Persistent forgetfulness^1^ (%)	23.5	26.3	0.796
Persistent sleep disturbances^1^ (%)	17.6	21.1	0.747
Persistent headaches^1^ (%)	17.6	13.2	0.707
Days hospitalized during acute COVID‐19	–	18.7 ± 15.7	

MoCA, Montreal Cognitive Assessment; HADS‐A/D, Hospital Anxiety Depression Scale; FAS, Fatigue Assessment Scale. Data are expressed as mean ± standard deviation, when not otherwise specified. Mean comparison was performed using independent *t*‐test or Kruskal–Wallis test according to data distribution. Significant *p* values (<0.05) are written in bold.

^1^ Self‐reported symptoms.

All participants performed three eye tracking tasks: overlapping pro‐saccade, anti‐saccade, dual‐task anti‐saccade. Eye tracking was performed using a Tobii TX300 system (Tobii Technology AB), showing visual stimuli on a 23 inches screen with a 1980 × 1280 pixels resolution. Participants sat in a quiet and dimly lighted room, on a comfortable armchair with a headrest to minimize involuntary head movements and to keep a constant distance of 65 cm from the computer screen. To maximize standardization, all participants were tested by the same investigator (FC) and all eye tracking tasks were presented in a predefined order.

1. In the overlapping pro‐saccade task, the cue and target were displayed on the screen simultaneously. Participants were instructed to perform a saccade toward the cue as soon as the target disappears. Saccades performed while the target is still present on the screen were considered errors. This task was repeated 80 times.
2. The anti‐saccade task is cognitively more demanding than a pro‐saccade. Subjects were asked to perform a saccade away from the peripheral cue. Saccades toward the cue were considered errors. The task was divided into two blocks of 30 repetitions each, with a short break in between.
3. In the dual task, the anti‐saccade was performed in concomitance with a motor task which consists of pressing a button on the computer keyboard on the same side as the peripheral cue appears on the monitor. This conflicting task leads to a worsening of the performance of the anti‐saccade; the decrement in performance (both in reaction time and error rate) is defined as “dual‐task cost” (DTC). DTC was calculated using the formula $100∙\left(x_{\text{dual}}-x_{\text{single}}\right)/x_{\text{single}}$, with x being reaction time, directional error, or error rate.

**Figure 1:**
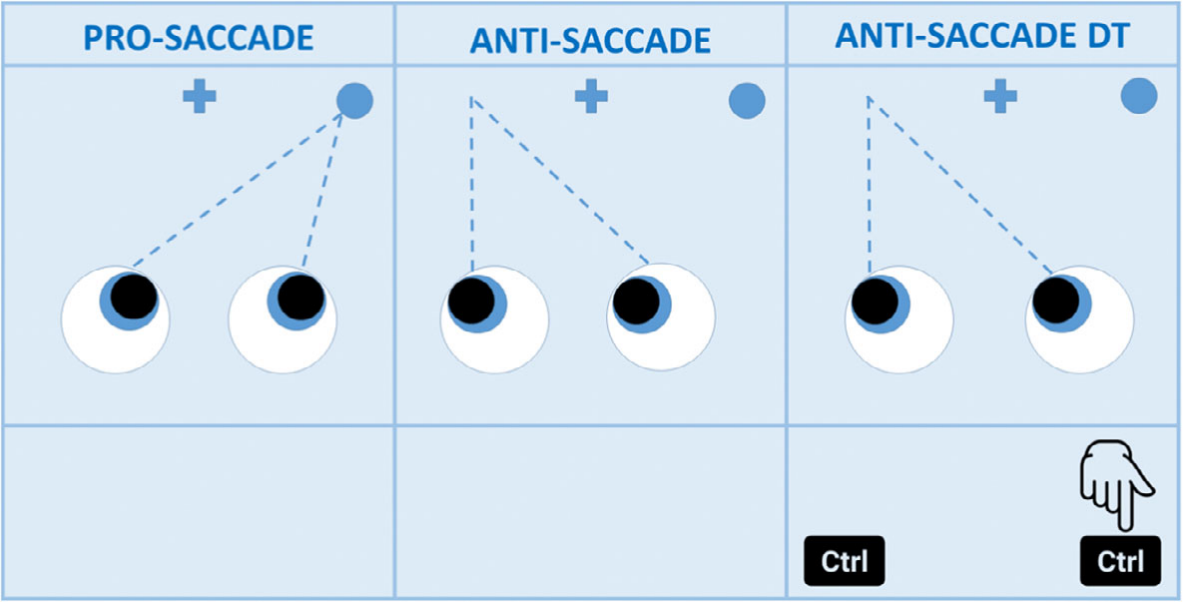
Participants are instructed to look at the fixation cross at the beginning of every task. In the pro‐saccade task they will look at the peripheral cue (dot); in the anti‐saccade task in the opposite direction; in the dual‐task (DT) participants are asked to press a computer key on the same side of the cue while they perform an anti‐saccade. For each task reaction time and error rate are recorded.

For each task, reaction time was measured from the appearance of the peripheral cue until the first saccade; the threshold under which saccades were discarded was 50 msec. Prior to each of the three tasks, participants performed a practice run consisting of four repetitions; verbal feedback was given when necessary. Between tasks, a break of 2 min was allowed. More details on the saccadic paradigms in the supplemental material, for a visual representation Figure 1.

### Statistical analysis

The statistical analysis was carried out using SPSS (v24). Based on data distribution, we employed parametric (independent *t*‐test) and non‐parametric (Mann–Whitney *U*, Kruskal–Wallis) tests to assess differences across groups. Bonferroni correction was used as multiple‐comparison correction. Age, MoCA scores, and education were used as covariates for all analyses. Correlation analysis was carried out using Spearman's analysis. A multiple regression analysis was used to predict error rates in saccadic tasks from independent variables. The level of significance for all analysis was set at *p* = 0.05.

## Results

### Demographics and disease characteristics

Demographic characteristics are summarized in Table 1.

### Saccadic tasks

The results from saccadic tasks are reported in Table 2. Patients made more directional errors in the anti‐saccade task and in the dual‐task anti‐saccade task (*p* < 0.001 and *p* = 0.045, respectively) and more anticipation errors in the pro‐saccade task (*p* = 0.003) compared to HC. There were no differences in reaction time in any of the saccadic tasks (all *p‐*values >0.1). There were also no significant intergroup differences in DTC in regards to directional error, saccade not being performed, and reaction time (all *p*‐values >0.1). A correlation analysis showed no correlation between FAS score and performance on any saccadic paradigm (all *p*‐values >0.2).

**Table 2:** Results and comparison of saccadic performance among subgroups.

	Healthy controls (*n* = 23)	Outpatients (*n* = 17)	Inpatients (*n* = 38)	*p* value	H/I	H/O	I/O
Saccadic tasks parameters							
Pro‐saccade							
Anticipatory error	11.6 ± 9.7	26.9 ± 21.5	26.6 ± 24.8	**0.013**	**0.024**	**0.040**	0.989
Reaction time	283.8 ± 92.1	261.7 ± 43.9	302.4 ± 83.5	0.226	–	–	–
Anti‐saccade							
Directional error	8.9 ± 6.1	13.4 ± 12.9	24.1 ± 17.5	**0.001**	**0.001**	0.999	**0.026**
Saccade not performed	0.8 ± 1.5	0.9 ± 2.4	2.8 ± 4.6	0.084	–	–	–
Reaction time – Wrong	194.1 ± 69.1	211.7 ± 85.2	210.0 ± 50.0	0.293	–	–	–
Reaction time – Correct	275.6 ± 80.4	254.3 ± 18.7	304.9 ± 68.6	**0.004**	**0.030**	0.825	**0.005**
Anti‐saccade Dual task							
Directional error	21.4 ± 9.7	23.1 ± 18.6	38.9 ± 23.1	**0.006**	**0.033**	1.000	**0.022**
Saccade not performed	1.9 ± 2.1	0.9 ± 2.1	5.8 ± 12.9	0.429	–	–	–
Reaction time – Wrong	243.7 ± 93.2	215.1 ± 64.2	239.8 ± 57.4	0.220	–	–	–
Reaction time – Correct	342.6 ± 107.6	297.2 ± 65.4	384.0 ± 110.7	**0.014**	0.289	0.721	**0.014**
Dual‐task cost							
Directional error	12.5 ± 10.1	9.6 ± 10.3	14.9 ± 15.7	0.513	–	–	–
Saccade not performed	1.1 ± 1.9	1.8 ± 4.1	8.7 ± 12.7	0.429	–	–	–
Reaction time – Correct	66.5 ± 53.5	42.8 ± 57.2	79.1 ± 79.5	0.138	–	–	–

Significant *p* values <0.05 are reported in bold. Samples were compared using Mann–Whitney *U* test. We report the adjusted significance using Bonferroni correction. Errors are expressed as percentage, reaction time in milliseconds. Dual‐task cost is expressed as percentage and calculated with the formula: ((dual‐task single task)/single task × 100). H, healthy controls; I, inpatients; O, outpatients. Significant *p* values (<0.05) are written in bold.

A sub‐analysis showed that inpatients made significantly more directional errors in the anti‐saccade task compared to outpatients (*p* = 0.03) and HC (*p* = 0.001). Outpatients had a similar error rate to HC (*p* > 0.1). Additionally, inpatients had longer reaction times in the correctly performed anti‐saccade task compared to both outpatients (*p* = 0.005) and HC (*p* = 0.03). Inpatients made also more directional errors in the dual‐task anti‐saccade paradigm compared to outpatients (*p* = 0.02) and HC (*p* = 0.03) and had longer reaction times in correct trials than outpatients (*p* = 0.01). Finally, HC made fewer anticipatory errors in the pro‐saccade task compared to inpatients (*p* = 0.02) and outpatients (*p* = 0.04) but there was no difference between the 2 patient subgroups (*p* > 0.6). The difference in performance in the three saccadic tasks remained significant after controlling for age, MoCA scores, and education (*Wilks' Lambda* = 0.798, *p* = 0.030). A Spearman's analysis showed no significant correlation between MoCA scores and saccadic performance (*p* > 0.2 for all subgroups). Additionally, there was no correlation between saccadic performance and subscores of the MoCA (*p* > 0.3).

## Discussion

Here, we assessed cognitive function in COVID‐19 patients compared to HC using eye tracking 12 months after disease onset. We found that patients made significantly more directional errors on the anti‐saccade and on the dual task and also made more anticipatory errors in the pro‐saccade task. Furthermore, those patients requiring hospitalization performed worse than HC and patients who were managed as outpatients. Specifically, inpatients were slower and made more directional errors in the dual task and in the anti‐saccade task than HC and outpatients and made more anticipatory errors compared to HC.

Given the literature, we used a targeted approach to evaluate executive function, specifically response inhibition, attention, and working memory.^8^ These cognitive resources are necessary for the anti‐saccade task, which requires intact top‐down inhibition systems, intact frontal areas (such as frontal eye fields and dorsolateral prefrontal cortex), and is mediated by the basal ganglia.^10^ Our results show that these functions are impaired in patients even 1 year after COVID‐19. Error rates described here are comparable to those of patients diagnosed with various neurological/psychiatric diseases affecting frontal lobes and/or basal ganglia.^11^, ^12^, ^13^ This impairment in saccadic inhibition is also reflected by the performance in the overlap pro‐saccade task, where patients were unable to effectively delay an automatic saccade.

We found no correlation with MoCA subscores and saccadic performance, possibly due to a ceiling effect. The MoCA is a rapid cognitive screening tool, and our patients had normal scores.

One recent study assessed eye movement abnormalities in patients post‐COVID‐19 and found abnormalities in latencies of various tasks. However, the sample size (*n* = 9) is too low to interpret the results. Our main results were obtained using a standardized anti‐saccade protocol, allowing for comparison with existing and future studies.^14^

Our results are in line with a recent 18fluorodeoxyglucose (^18^F‐FDG) PET study showing a correlation between COVID‐19 symptoms' severity and frontoparietal hypometabolism; they also agree with other functional imaging studies in patients suffering from “Long COVID” showing hypometabolism in the cingulate cortex, right temporal lobe, and cerebellum: all areas involved in the cognitive domains of attention, memory, and executive function.^2^, ^15^, ^16^

Task‐related fatigue did not affect saccadic performance, as there was no difference between errors in the first and second half of the saccadic tasks. Additionally, there was no correlation between reported fatigue and saccadic performance. We cannot exclude a learning effect since all tasks were repeated in the same order; this was nevertheless necessary to perform the dual‐task paradigm. Also, we cannot exclude that some of the HC might have had an asymptomatic Sars‐CoV‐2 infection.

The executive dysfunctions described in this study are not specific for COVID‐19 and executive functions are not the only cognitive domains affected in these patients. Similar impairments have been described in patients with Severe Acute Respiratory Syndrome and Middle East Respiratory Syndrome, during the active phase of disease but also after physical recovery.^17^

In conclusion, this cross‐sectional controlled study showed an impairment in executive functions in patients 1 year after COVID‐19, observed for the first time using eye tracking assessments. This objective approach highlights a greater impairment in those who were treated as inpatients. Our results underline a significant and under‐recognized condition that should be assessed and addressed, as it may have important repercussions in patients' everyday activities. COVID‐19 survivors, especially older and hospitalized patients, should be followed up with neuropsychiatric and neuropsychological assessments according to clinical indication, as they may benefit from tailored mental health and cognitive rehabilitation programs.

## Conflict of Interest

KS reports grants from FWF Austrian Science Fund, grants from Michael J. Fox Foundation, grants from International Parkinson and Movement Disorder Society, personal fees from Teva, personal fees from UCB, personal fees from Lundbeck, personal fees from AOP Orphan Pharmaceuticals AG, personal fees from Abbvie, personal fees from Roche, personal fees from Grünenthal; all outside the submitted work. PM reports grants from TWF (Tyrolean Science Fund), grants from Medtronic, personal fees from Boston Scientific, all outside the submitted work. The other authors have nothing to disclose and declare no competing interests.

## Authors Contributions

Conceptualization of the study: AD, FC. Funding acquisition: VR, RH. Supervision: AD. Patient samples and data collection: FC, SB, LZ, VR. Data analysis and curation: FC, AD. Methodology: AD, FC, MR, PE. Visualization: AD, FC. Writing the original draft: FC. Review and editing of the manuscript: AD, RH, KS, SK, JLR, PK, LP, BP, AL, MK, AJS, PE, MP, VL, BH, PM, RB, MR, SB, VR, LZ.

## Supporting information


**Appendix S1.** Supplementry material.

## Data Availability

Raw data were generated at the Medical University of Innsbruck, Department of Neurology. Derived data supporting the findings of this study are available from the corresponding author (A.D.) on request.

## References

[1] Helms J , Kremer S , Merdji H , et al. Neurologic features in severe SARS‐CoV‐2 infection. N Engl J Med. 2020;382:2268‐2270. 32294339 10.1056/NEJMc2008597 PMC7179967

[2] Hosp JA , Dressing A , Blazhenets G , et al. Cognitive impairment and altered cerebral glucose metabolism in the subacute stage of COVID‐19. Brain. 2021;144:1263‐1276. 33822001 10.1093/brain/awab009 PMC8083602

[3] Tavares‐Júnior JWL , de Souza ACC , Borges JWP , et al. COVID‐19 associated cognitive impairment: a systematic review. Cortex. 2022;152:77‐97. 35537236 10.1016/j.cortex.2022.04.006 PMC9014565

[4] Graham EL , Clark JR , Orban ZS , et al. Persistent neurologic symptoms and cognitive dysfunction in non‐hospitalized Covid‐19 “long haulers”. Ann Clin Transl Neurol. 2021;8:1073‐1085. 33755344 10.1002/acn3.51350 PMC8108421

[5] Evans RA , McAuley H , Harrison EM , et al. Physical, cognitive, and mental health impacts of COVID‐19 after hospitalisation (PHOSP‐COVID): a UK multicentre, prospective cohort study. Lancet Respir Med. 2021;9:1275‐1287. 34627560 10.1016/S2213-2600(21)00383-0 PMC8497028

[6] Liu L , Ni SY , Yan W , et al. Mental and neurological disorders and risk of COVID‐19 susceptibility, illness severity and mortality: a systematic review, meta‐analysis and call for action. EClinicalMedicine. 2021;40:101111. 34514362 10.1016/j.eclinm.2021.101111 PMC8424080

[7] Daroische R , Hemminghyth MS , Eilertsen TH , Breitve MH , Chwiszczuk LJ . Cognitive impairment after COVID‐19—a review on objective test data. Front Neurol. 2021;12:699582. 34393978 10.3389/fneur.2021.699582 PMC8357992

[8] Duan K , Premi E , Pilotto A , et al. Alterations of frontal‐temporal gray matter volume associate with clinical measures of older adults with COVID‐19. Neurobiol Stress. 2021;14:100326. 33869679 10.1016/j.ynstr.2021.100326 PMC8041745

[9] Rass V , Beer R , Schiefecker AJ , et al. Neurological outcome and quality of life 3 months after COVID‐19: a prospective observational cohort study. Eur J Neurol. 2021;28:3348‐3359. 33682276 10.1111/ene.14803 PMC8250725

[10] Coe BC , Munoz DP . Mechanisms of saccade suppression revealed in the anti‐saccade task. Philos Trans R Soc B Biol Sci. 2017;372:20160192. 10.1098/rstb.2016.0192 PMC5332851 28242726

[11] Zee DS , Lasker AG . Antisaccades: probing cognitive flexibility with eye movements. Neurology. 2004;63:1554. 15534233 10.1212/01.wnl.0000142978.08638.df

[12] Barbosa P , Kaski D , Castro P , Lees AJ , Warner TT , Djamshidian A . Saccadic direction errors are associated with impulsive compulsive Behaviours in Parkinson's disease patients. J Parkinsons Dis. 2019;9:625‐630. 31282422 10.3233/JPD-181460

[13] Carbone F , Ellmerer P , Ritter M , et al. Impaired inhibitory control of saccadic eye movements in cervical dystonia: an eye‐tracking study. Mov Disord. 2021;1–5:1246‐1250. doi:10.1002/mds.28486 PMC8247854 33416199

[14] Antoniades C , Ettinger U , Gaymard B , et al. An internationally standardised antisaccade protocol. Vision Res. 2013;84:1‐5. 23474300 10.1016/j.visres.2013.02.007

[15] Guedj E , Campion JY , Dudouet P , et al. 18F‐FDG brain PET hypometabolism in patients with long COVID. Eur J Nucl Med Mol Imaging. 2021;48:2823‐2833. 33501506 10.1007/s00259-021-05215-4 PMC7837643

[16] Hugon J , Msika EF , Queneau M , Farid K , Paquet C . Long COVID: cognitive complaints (brain fog) and dysfunction of the cingulate cortex. J Neurol. 2021;2–4:44‐46. doi:10.1007/s00415-021-10655-x PMC8211714 34143277

[17] Zhou H , Lu S , Chen J , et al. The landscape of cognitive function in recovered COVID‐19 patients. J Psychiatr Res. 2020;129:98‐102. 32912598 10.1016/j.jpsychires.2020.06.022 PMC7324344

