# Supplementary material for: Cognitive dysfunction 1 year after COVID‐19: evidence from eye tracking
Source: Ann Clin Transl Neurol. 2022 Oct 20;9(11):1826–31. doi: 10.1002/acn3.51675 (PMC9639623; doi:10.1002/acn3.51675)
Supplement: Supplementary file 1 — Appendix S1. Supplementry material. [file ACN3-9-1826-s001.docx]

**Supplementary material**

**Methods**

**HADS-D scoring:** This scale consists of a subscale for depression and one for anxiety; scores >7 imply borderline cases and scores > 10 are suggestive of depression and anxiety.

**Saccadic Paradigms:**

Raw eye movement data were acquired with a 300 Hz sampling rate and exported through a dedicated software (Tobii Pro Lab, version 1.83). Before every test a 9-point calibration was performed, in order to obtain a gaze position accuracy under 0.4 degrees.

Pro-saccade task: the target presented is a white cross (dimensions 0.5 x 0.5 degrees) on a black background for a random period of time of either 800ms or 1200ms. Introducing this variability at the beginning of the trial prevents the subject from getting unconsciously used to the test structure and possibly increasing the number of premature automated saccades to cue in the following part. The cue is a white point (radius 0.5 degrees), appearing 10 degrees to the right or to the left of the cross. The overlapping phase lasts randomly either 300ms or 700 ms, afterwards the cue stays on the screen 1000 ms. This task is repeated 80 times in one block.

Anti-saccade task/Anti-saccade dual task: the same target and cue used in the pro-saccade task are employed. The central target stays on the screen for 1500 ms and is then simultaneously replaced by the peripheral cue, appearing randomly 10 degrees to the right or to the left for 1000 ms.

**Results**

**Demographics and disease characteristics**

We found no significant differences in gender, age, education and MoCA scores between patients and HC (p>0.2). Twenty-eight (51%) patients reported having at least one persistent symptom one year after COVID-19, including fatigue (41%), difficulties in concentration (29%), forgetfulness (27%), sleep disturbances (21%), and headaches (14.5%). Patients were divided into those requiring hospital admission (*n*=38) and those who were managed as outpatients (*n*=17). We found that inpatients were more commonly males (male sex 71.0%) whereas most of the outpatients were females (male sex 23.6%, *p*=0.001). In addition, inpatients were older (mean age 58.1 vs 46.3, *p*=0.003) and had lower MoCA scores (mean scores 26.5 vs 28.43, *p*=0.013). Comparisons between HC and the two subgroups of patients revealed no significant difference in MoCA scores (*p*>0.1) age (*p*>0.08), gender (*p*>0.1), and education (*p*>0.08). The FAS revealed that 47% of the patients suffered from substantial fatigue at the time of the visit, without differences between inpatients and outpatients (*p*=0.1). A total of 5 (9.1%) and 4 (7.3%) patients had scores above the cut-off for anxiety and depression, respectively.

**Saccadic tasks**

A within-group analysis showed that all three subgroups performed worse on the dual-task anti-saccade compared to the standard anti-saccade paradigm: HC worsened from 8.9 ±6.1 to 21.4±9.7; outpatients from 13.4±12.9 to 23.1±18.6; inpatients from 24.1±17.5 to 38.9±23.1 (mean percentage error ± standard deviation, *p* for all subgroups <0.001).

A multiple regression was run to predict the error rate in the anti-saccade task from disease severity (healthy, inpatient, or outpatient), age, years of education, sex, and MoCA score. These variables significantly predicted the error rate (*p*<0.001, *R^2^* =0.327) but only disease severity and advanced age added significantly to the prediction (*p*=0.007, *p*=0.029 respectively).
